# Hydrosols from *Rosmarinus officinalis*, *Salvia officinalis*, and *Cupressus sempervirens*: Phytochemical Analysis and Bioactivity Evaluation

**DOI:** 10.3390/plants11030349

**Published:** 2022-01-27

**Authors:** Matteo Politi, Claudio Ferrante, Luigi Menghini, Paola Angelini, Giancarlo Angeles Flores, Beatrice Muscatello, Alessandra Braca, Marinella De Leo

**Affiliations:** 1 Dipartimento di Farmacia, Botanic Garden “Giardino dei Semplici”, Università di Chieti-Pescara, Via Vestini 1, 66100 Chieti Scalo, Italy; matteo.politi@unich.it (M.P.); claudio.ferrante@unich.it (C.F.); luigi.menghini@unich.it (L.M.); 2 Dipartimento di Chimica, Biologia e Biotecnologia, Università di Perugia, Via del Giochetto 6, 06122 Perugia, Italy; paola.angelini@unipg.it (P.A.); giancarlo.angelesflores@studenti.unipg.it (G.A.F.); 3 Dipartimento di Farmacia, Università di Pisa, Via Bonanno 33, 56126 Pisa, Italy; beatrice.muscatello@unipi.it (B.M.); marinella.deleo@unipi.it (M.D.L.); 4 Centro per l’Integrazione della Strumentazione dell’Università di Pisa (CISUP), Lungarno Pacinotti 43, 56126 Pisa, Italy

**Keywords:** *Rosmarinus officinalis*, *Salvia officinalis*, *Cupressus sempervirens*, aromatic water, hydrosol, hydrolate, fingerprinting analysis, cytotoxicity, in silico analysis

## Abstract

The present work evaluates the aromatic waters of rosemary (*Salvia rosmarinus* Spenn. syn. *Rosmarinus officinalis* L.), sage (*Salvia officinalis* L.), and cypress (*Cupressus sempervirens* L.) obtained as innovative commercial products of a hydrodistillation process. All extracts were exhaustively analysed by GC-MS, ^1^H-NMR, and LC-MS in order to evaluate potential metabolite fingerprint differences. GC-MS appears to be the most exhaustive technique for the qualitative identification of the single constituents, although in this case, the use of ^1^H-NMR and LC-MS techniques allowed some useful considerations in semi-quantitative terms. Antimycotic effects were studied against *Tricophyton*, *Candida*, and *Arthroderma* species, resulting in weak activity. The toxicological impact was partly evaluated in vitro by means of allelopathy and brine shrimp lethality. Cytotoxicity was investigated in human colon cancer cells (HCT116) and in hypothalamic cells (Hypo-E22) challenged with hydrogen peroxide. Sage and rosemary hydrosols were the most effective antimycotics, whereas all hydrosols displayed antiradical effects. Cytotoxic effects against HCT116 cells (at 500 µL/mL) were related in silico to the endovanilloid TRPM8 and TRPV1 receptors. At lower concentrations (5–50 µL/mL), the hydrosols protected hypothalamic neurons Hypo-E22 cells from hydrogen peroxide-induced toxicity. The overall experience indicates that hydrolates are an important source of relevant phytochemicals with significant pharmacological potential.

## 1. Introduction

Aromatic waters, also known as hydrolates or hydrosols, are the aqueous phase obtained from the steam or hydrodistillation of different parts of aromatic plants, which separate from the essential oil phase at the end of the distillation process [1]. While the scientific literature on essential oils is relatively abundant, much less is known regarding aromatic waters [2]. Metabolite profiles of hydrolates may or may not qualitatively overlap with those of the corresponding essential oils, though they usually differ in quantitative terms. In a comparison between 44 hydrolates with the corresponding essential oils, it was found that in 42% of the cases, the main components of essential oils and hydrolates were different [3]. This trend was also observed in other cases [4,5,6], indicating the relevance of testing the biological activities of this kind of undervalued natural extract [7], which have been recently proposed as the main products of aromatic plant distillation [8].

It is known that the volatile profiles of hydrolates can vary depending not only on the origin of the plant materials but also on the applied analytical techniques and instrumental conditions used for the analysis [9]. Therefore, in this work, a full metabolomic platform based on GC-MS, LC-MS, and NMR techniques for the analysis of the aromatic waters obtained by hydrodistillation of the flowering aerial parts of rosemary (*Salvia rosmarinus* Spenn. syn. *Rosmarinus officinalis* L.) and sage (*Salvia officinalis* L.), as well as the cones of cypress (*Cupressus sempervirens* L.), was applied. Although these plants are well-known botanical species of the Mediterranean flora, literature data on their aromatic waters are rather scarce.

Borneole, camphor, 1,8-cineole, and verbenone appear to be the major compounds in the aromatic waters from rosemary grown in Corsica [6] and in Japan [10], although their relative amounts appear to be partially different; in fact, rosemary can be distinguished in different chemotypes including camphor, cineole, α-pinene, and verbenone types [11]. Although most of the biological data on rosemary and other aromatic waters focus on their antimicrobial activities [2,12,13], the potential beneficial properties on cognition and the cerebrovascular system of acute consumption of rosemary aromatic water on healthy adults have been recently assessed [14].

The aromatic water obtained from a sage sample collected and distilled in Turkey showed camphor, 1,8-cineole, α- and β-thujone, and borneole as major constituents [15]. Another aromatic water sample obtained from plant material collected in Morocco with a similar volatile profile showed poor antioxidant properties compared with other Mediterranean plant species hydrosols [16].

Despite the fact that the constituents of cypress cones have been investigated [17], as well as the corresponding essential oil profile [18], as far as we know, there are no literature data on the volatile constituents of the aromatic water of *C. sempervirens*; only the hydrosols of others *Cupressus* species, including *C. lusitanica* [19] and *C. atlantica* [20], were previously investigated.

Due to the low amount of literature data on the aromatic water from rosemary, sage, and cypress, the aim of the present work was to perform an in-depth phytochemical analysis based on headspace, solid phase microextraction coupled with gas chromatography-mass spectrometry (HS-SPME-GC-MS), ultra-high performance liquid chromatography coupled to a high-resolution mass spectrometry (UHPLC-HR-MS), and nuclear magnetic resonance (NMR) analyses. Moreover, ecotoxicological, antimycotic, cytotoxic, and protective effects induced by the aromatic waters were also investigated in different in vitro models, whereas a bioinformatics analysis was conducted to identify putative protein targets underlying the observed effects. The results supported the rationale for the pharmaceutical use of the present hydrosols.

## 2. Results

### 2.1. HS-SPME-GC-MS of the Hydrosols

Sampling of the volatile organic compounds (VOCs) in the headspace of the aromatic water was carried out by the SPME technique, followed by GC-MS analysis (Figures S1–S3 in Supplementary Materials). Table 1 lists all the identified compounds. The identification was obtained by comparing retention times with those of pure compounds, linear relative indices (LRI) to a series of *n*-hydrocarbons, and mass spectra against commercial (NIST 98 and ADAMS) and homemade libraries of mass spectra.

Oxygenated monoterpenes represented the most abundant class in all samples. In rosemary’s hydrosol headspace, 70.4% of the compounds found were oxygenated monoterpenes, mainly 1,8-cineole (47.1%), camphor (5.4%), borneol (3.7%), verbenone (2.8%), and linalool (2%). Among the monoterpene hydrocarbons, camphene (3.9%), β-pinene (3.6%), and α-pinene (1.4%) were the most abundant, as in the essential oil composition [21,22,23]. 

In sage’s hydrosol headspace, oxygenated monoterpenes accounted for 93.7% of the total VOC content, with the predominance of 1,8-cineole (42.9%), α-thujone (24.3%), β-thujone (14.7%), and camphor (8.9%). The main components of the monoterpene and sesquiterpene hydrocarbons were β-pinene and β-caryophyllene, respectively. As reported in the literature, these compounds were also the major components in the essential oil from sage [24,25,26].

The aromatic water obtained from cypress cones contained 73.8% of oxygenated monoterpenes, mainly 4-terpineol (44.5%) and α-terpinyl acetate (10.6%), 23% of monoterpene hydrocarbons with 4.9% of δ-3-carene, 4.5% of α-pinene, and 2.4% of both sabinene and limonene and a small content of sesquiterpene hydrocarbons (cedrene, β-caryophyllene, and germacrene D). In this case, the composition was quite different from the essential oil, which is mainly rich in α-pinene, 1,8-cineole, camphor, borneol, and α-terpineol [27,28,29].

### 2.2. ^1^H-NMR and UHPLC-HR-MS Analyses of the Hydrosols

**Figure 1:**
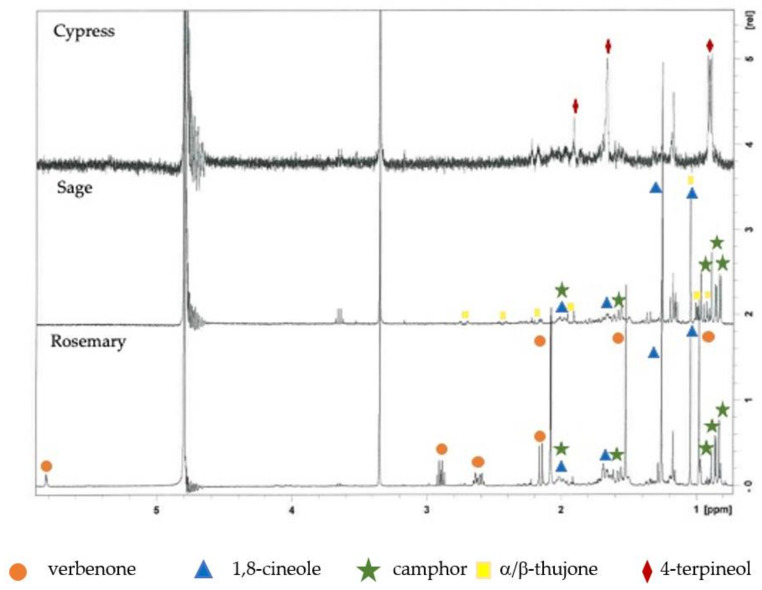
^1^H-NMR spectra of rosemary, sage, and cypress hydrolates.

In accordance with our previous work [8], ^1^H-NMR spectra with the suppression of the water signal were carried out for the hydrolate analysis and reported in Figure 1. Rosemary hydrosol spectrum showed the presence of signals attributable to verbenone (δ 5.82, 2.88, 2.63, 2.17, 2.08, 1.52, and 0.98), 1,8-cineole (δ 2.08, 1.66, 1.61, 1.26, and 1.05) and camphor (δ 2.08, 1.53, 0.99, 0.90, and 0.85); sage hydrolate displayed signals for 1,8-cineole (δ 2.05, 1.66, 1.59, 1.26, and 1.05), camphor (δ 2.02, 1.55, 0.97, 0.90, and 0.85), and, in a lesser amount, α/β-thujone (δ 2.72, 2.44, 2.16, 1.05, 0.98, and 0.93), while ^1^H-NMR spectrum of cypress hydrosol showed few signals, assignable only to 4-terpineol (δ 1.91, 1.67, 0.92, and 0.90) [30,31,32,33,34].

**Figure 2:**
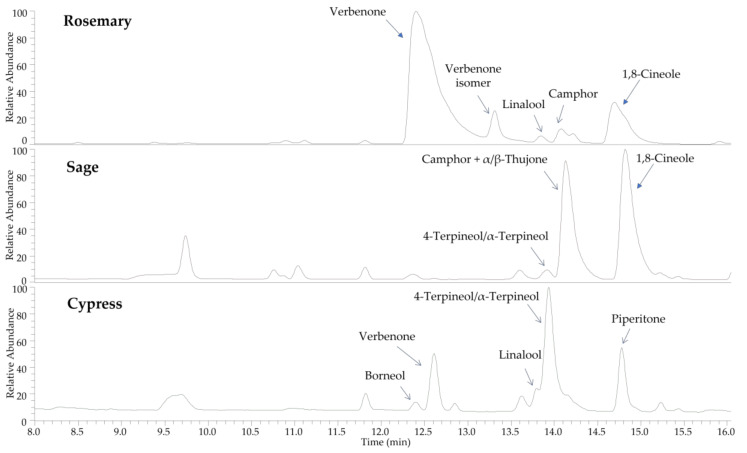
Chemical profiles (total ion current) of rosemary, sage, and cypress hydrolates obtained by UHPLC-HR-ESI-MS analysis.

The chemical profiles of rosemary, sage, and cypress hydrolates, investigated by means of UHPLC-HR-MS, are reported in Figure 2.

In all hydrolates, the major components were represented by volatile compounds, according to results obtained by GC-MS analyses, while no phenolics or other non volatile constituents were recorded. In particular, oxygenated monoterpenes were found, such as verbenone, borneol, linalool, 4-terpineol, α-terpineol, camphor, 1,8-cineole, and piperitone. The distribution of compounds in the hydrolates, together with MS data registered in positive ion mode, are reported in Table 2.

All molecules were identified by comparison of full and fragmentation mass spectra with those of reference standards, except for piperitone, which was tentatively attributed by analysis of MS/MS data. In full MS experiments, the monoterpene alcohols borneol, linalool, 4-terpineol, α-terpineol, and 1,8-cineole showed protonated molecular ions at *m*/*z* 137.1323 as precursor ions after the loss of a water molecule ([M+H-H_2_O]^+^ in the ESI source). These molecules also showed very similar fragmentation pathways; thus, the comparison of retention times with reference standards was useful for their discrimination. Camphor, α/β-thujone, and piperitone are monoterpene ketones showing in the full ESI-MS the same protonated molecular ion at *m*/*z* 153.127. Fragmentation experiments generated very similar product ions, but with different abundance (Table 2), which could be diagnostic for the differentiation of the three components. Some small peaks remained unidentified.

### 2.3. Biological Studies of the Hydrosols

#### 2.3.1. Allelopathy Test

**Figure 3:**
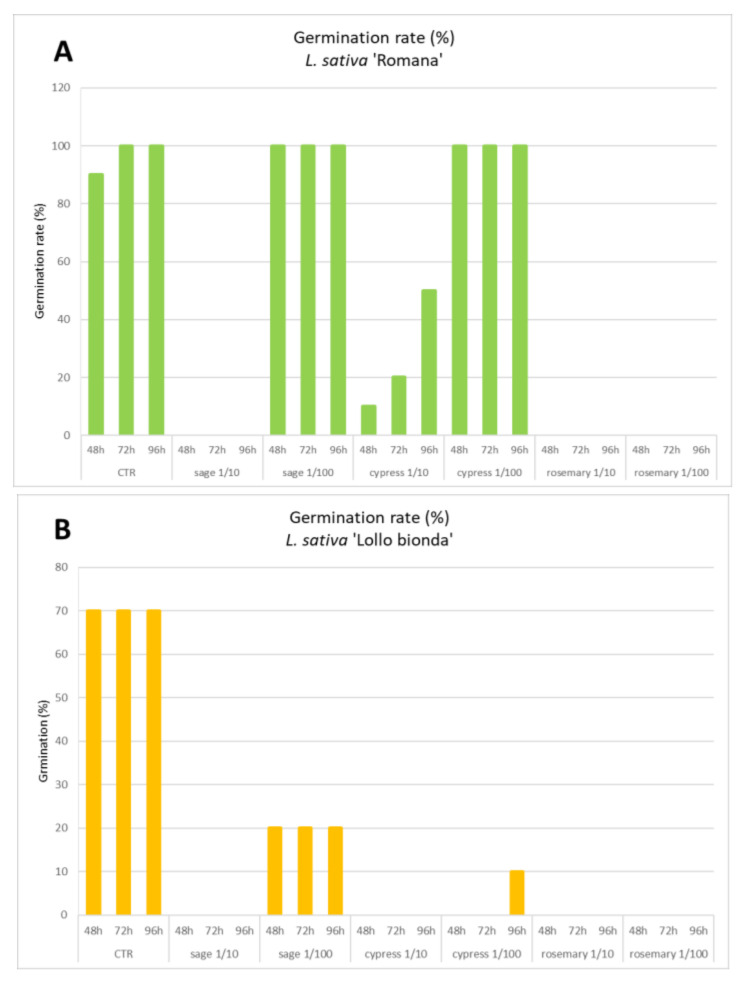

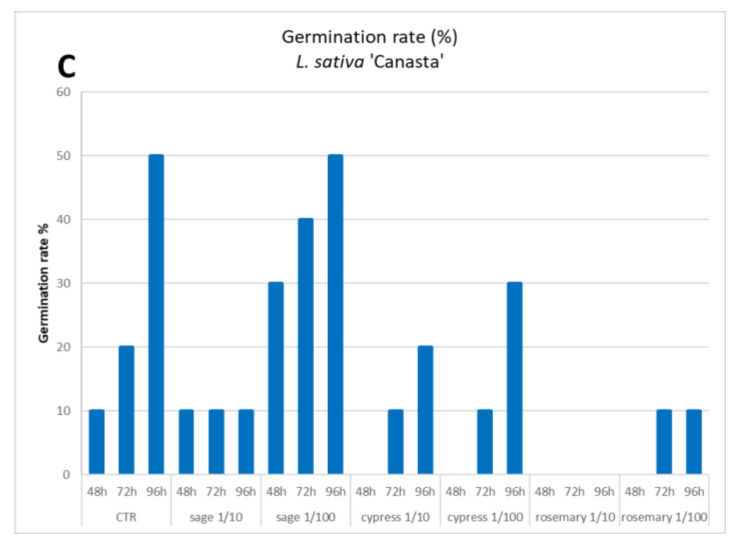
Allelopathic effects of supplementation of hydrosols in the soaking water used for seed germination of *L. sativa* varieties, namely Romana, Lollo Bionda, and Canasta (panels (**A**–**C**), respectively). Effects were monitored at 48, 72, and 96 h and the germination percentage was registered in the control (CTR, distilled water) and treated groups with two hydrosol dilutions. Data are mean values of four independent experiments.

The effects of hydrosol dilution on the seed germination of three lettuce varieties are reported in Figure 3. The allelopathy test revealed intrinsic differences related to the physiology of the germination process, as well as sensitivity to the treatment between the different tested varieties. The variety Canasta was characterized by delayed germination; after 4 days, only half of the tested seeds showed emergent radicles while being less sensitive to the presence of hydrosols in the soaking solution. Only the rosemary hydrosol at the highest tested concentration completely inhibited the germination of the Canasta variety and other varieties as well. The germination of the Romana variety was inhibited by all hydrosols with 0% germination in the presence of sage or rosemary. Weaker inhibition was due to the presence of cypress that reduced the germination from 90–100% of the control to 10–50%. At higher dilution (1/100), sage and cypress lost their inhibitory effect (100% germination), while rosemary was still completely active (0%). The Lollo Bionda variety was the most sensitive to the presence of hydrosols, with complete or strong germination inhibition (germination range 0–20%), which was revealed for all tested samples at all dilutions. The hydrosol obtained from sage distillation was the most biocompatible, at least if applied diluted 100 fold, with no or partial effects on germination rate. Conversely, the rosemary hydrosol revealed a strong and broad spectrum of inhibitory effects on seed germination. These effects suggested cautions in the use of this extract without an appropriate dilution while remaining open to innovative strategies to investigate non-food applications, such as selective herbicides.

#### 2.3.2. Brine Shrimp Lethality Test

**Figure 4:**
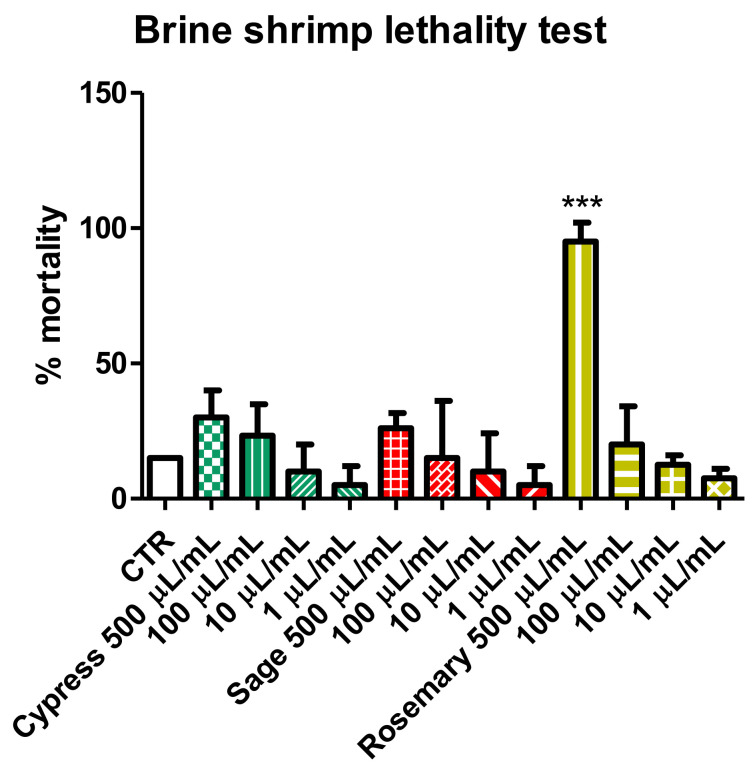
Brine shrimp lethality test. Effects of supplementation of hydrosols in artificial water used to breed newborn *Artemia salina*. Effects were monitored after 24 h treatment, and the mortality percentage was recorded in treated and control (CTR-artificial seawater) groups. Data are mean values ± S.D. of triplicate experiments. ANOVA, *p* < 0.001; *** *p* < 0.01 vs. control untreated group.

The potential toxic effects of the hydrosols added to the medium were evaluated in the ecotoxicological context using *Artemia salina* newborns. After a 24 h treatment, the effects of different concentrations of tested samples were evaluated and expressed as percentage mortality compared to the control seawater group. Data reported in Figure 4 showed that hydrosols are biocompatible at 100 µL/mL compared to the artificial seawater group. Cypress and sage showed a limited increase of mortality (mean value 30 and 26%, respectively) at higher concentrations, although this effect was not statistically significant compared to the control group. Only the rosemary hydrosol highlighted potential toxicity (mean mortality 95%), although at the highest tested concentration (500 µL/mL), thus suggesting that a simple dilution of the product was sufficient to guarantee environmental safety. The present data agreed with the allelopathy test results, confirming the rosemary hydrosol as the most active.

#### 2.3.3. Antifungal Activity

The antifungal activity of the hydrosols is reported in Table 3; it is also presented in comparison with the reference drugs fluconazole and griseofulvin. Among the tested samples, the rosemary and sage hydrosols displayed the best antimycotic profile, with MIC values in the range of 7.81–62.5 µL/mL. The MIC values demonstrated an intrinsic weak antifungal activity compared to the reference drugs, while considering the dilution and the aqueous nature of the tested hydrosols, the detected activity could result in interest for a wide range of applications. Intriguingly, the strain sensitivity was similar, at least from a qualitative point of view. 

*Trichophyton erinacei* and *Arthroderma gypseum* were resistant to tested hydrosols, while all other strains were inhibited at concentrations lower than 100 µL/mL.

#### 2.3.4. Cell Viability and Neuroprotective Effect

**Figure 5:**
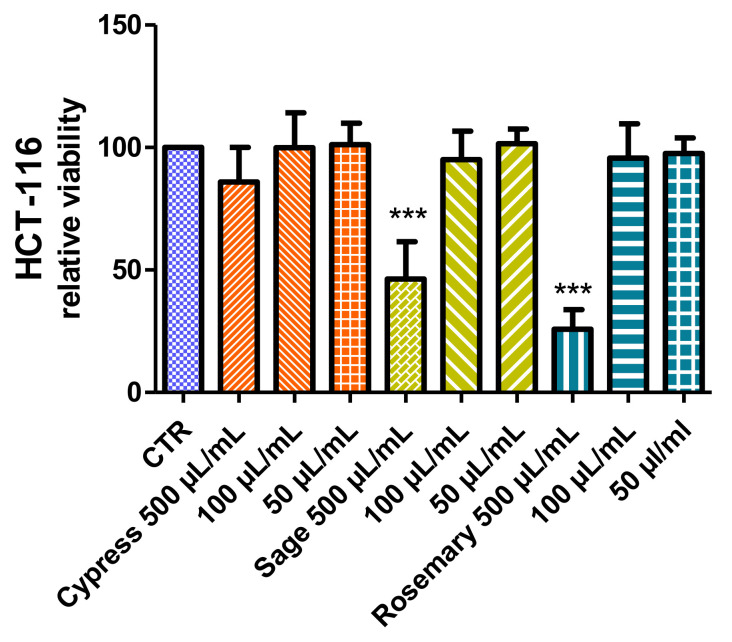
Anti-proliferative effects induced by the hydrosols. MTT assay was conducted on HCT116 cells exposed to hydrosols (50–500 µL/mL) for 24 h. ANOVA, *p* < 0.001; *** *p* < 0.01 vs. control untreated group.

The effects of the hydrosol supplementation of the culture media were tested on the colorectal cancer HCT116 cell line. As reported in Figure 5, after 24 h, only the highest concentration demonstrated alterations in cell viability, which was significantly reduced, at values lower than 50%, in the presence of rosemary and sage. No significant effect (within the range 85–101%) on cell viability was recorded at the lowest concentrations (100 and 50 µL/mL), thus supporting the good biocompatibility of the tested extracts. Noteworthy, the hydrosol from cypress was also well tolerated by cells at 500 µL/mL.

**Figure 6:**
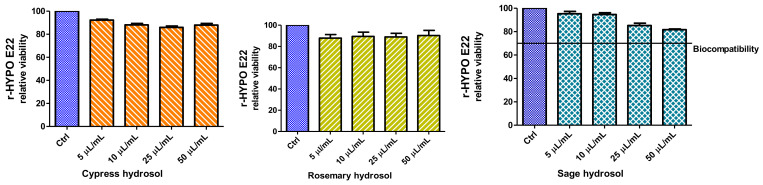
Null effect of hydrosols on hypothalamic neurons. MTT assay was conducted on hypothalamic Hypo-E22 cells exposed to the hydrosols (5–50 µL/mL) for 24 h.

In order to further explore potential biological effects, the biocompatibility of extracts was investigated on hypothalamic cells (Hypo-E22), which was also used as an experimental model for unravelling neuroprotective effects. Hydrosols were tested in the concentration range of 5–50 µL/mL, and no effect was detected on cell viability (Figure 6). Only the sage hydrosol showed a viability reduction that appeared concentration-dependent; however, even at the highest tested concentration, the percentage viability was higher than 70% compared to the control group (namely 81% at 50 µL/mL). This viability limit is commonly accepted as an index of biocompatibility.

**Figure 7:**
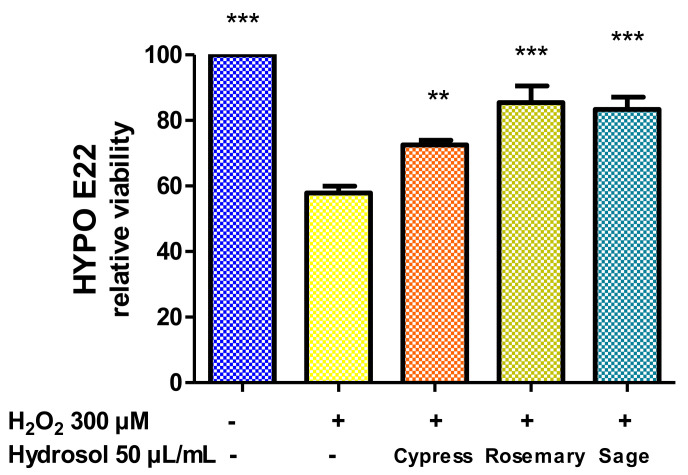
MTT assay of hypothalamic Hypo-E22 cells exposed to hydrosols (50 µL/mL) for 24 h challenged with 300 µM H_2_O_2_. ANOVA, *p* < 0.001; ** *p* < 0.01, *** *p* < 0.05 vs. control group.

From the present findings, the highest concentration (50 µL/mL) was considered well tolerated by Hypo-E22 cells and subsequently tested as a sub-toxic concentration to explore the protective effects against the neurotoxicity induced by exogenous stimulus (hydrogen peroxide 300 µM). Data in Figure 7 showed a strong reduction of cell viability induced by hydrogen peroxide. The effects can be partially but significantly reverted by preventive treatment with hydrosols, which were effective in contrasting and preventing the toxicity induced by the oxidative stimulus. In particular, rosemary and sage were more effective in blunting the hydrogen peroxide-induced toxicity, thus restoring the basal viability levels.

#### 2.3.5. Antioxidant Effect

In order to explore the intrinsic antioxidant activity of hydrosols, samples were tested for their ability to inhibit the substrate de-coloration induced by horseradish enzyme activated by hydrogen peroxide. The resulting data are reported in Table 4 and highlighted only a partial effect (19.53–31.58% inhibition).

#### 2.3.6. In Silico Analysis

**Figure 8:**
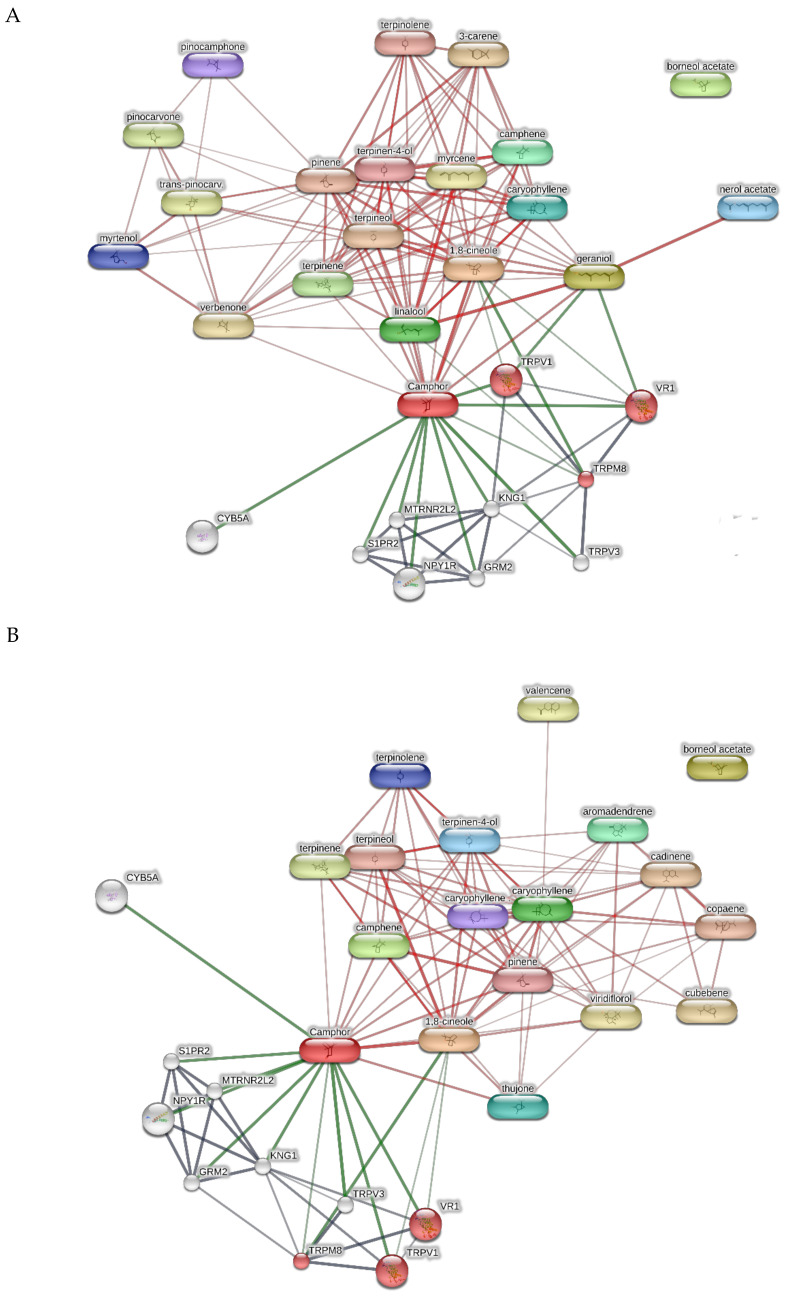

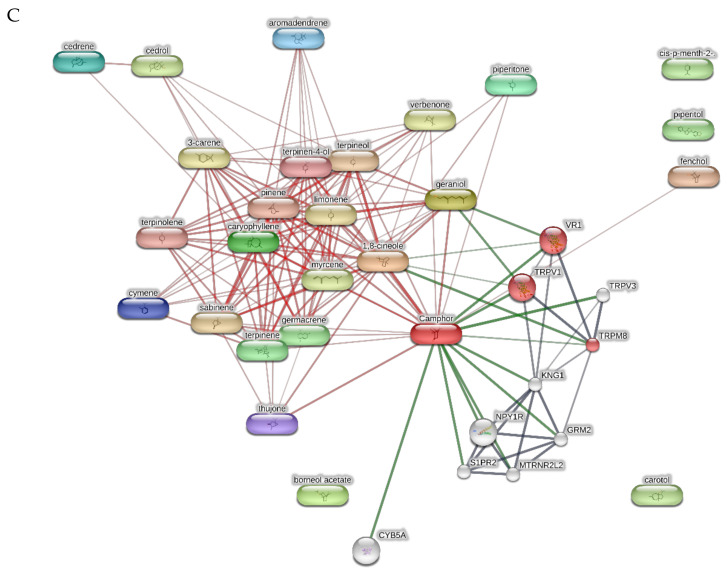
Target component analyses conducted on the bioinformatics platform STITCH for predicting putative proteins targeted by terpene compounds from the hydrosols of rosemary (**A**), sage (**B**) and cypress (**C**). The gene ontology analysis showed the probability of human thermoception pathway (GO: 0050955) modulation by different phytocompounds, including camphor, geraniol, 1,8-cineole, and fenchol.

Finally, the terpenes from hydrosols were also studied through the bioinformatics platform STITCH in order to predict putative proteins underlying the observed effects on cell viability. The target component and gene ontology analyses pointed to the modulation of endovanilloid receptors (thermoception pathway, GO: 0050955) by different terpene compounds, namely camphor, geraniol, 1,8-cineole, and fenchol (Figure 8).

## 3. Discussion

### 3.1. Phytochemical Analysis

Results obtained from the chemical analysis showed in all hydrolates the presence of more polar compounds, mainly oxygenated monoterpenes, compared with the other classes of VOCs; this is in accordance with our previous report [8]. On the basis of GC-MS data, 1,8-cineole (eucalyptol) appears to be the most abundant constituent in rosemary and sage extracts (47.1% and 42.9%), while 4-terpineol is the most abundant in the cypress hydrosol. In sage, the amount of α- and β-thujone is also relevant (24.3% and 14.7%). This is in agreement with the results obtained by NMR analysis, where the signals of these compounds are well detected in the corresponding spectra (Figure 1). The NMR data of rosemary hydrosol, however, also show a relevant amount of verbenone that appears comparable to 1,8-cineole, while in sage hydrosol, camphor appears in a comparable amount to α/β-thujone (both isomers are undistinguished via NMR analysis). Results obtained by LC-MS analyses confirmed NMR hydrolate fingerprints, especially regarding the most abundant compounds, even if some minor polar volatiles were also detected. Such semi-quantitative considerations are, therefore, in disagreement with the semi-quantitative data obtained via GC-MS, where verbenone in rosemary is 2.8%, while camphor is 5.4% and 8.9% in rosemary and sage, respectively. This evidence could be due to the different ionization of each molecule, which is known to be strictly related to its chemical structure. The efficacy of the MS-based methods for rapid semi-quantitative analysis of crude extracts such as the hydrosols here analyzed appears, therefore, lower compared to the NMR methods. This highlights the relevance of applying different spectroscopic methods in order to obtain a more exhaustive picture of the overall phytochemical fingerprint of the hydrosols. In this case, the NMR technique, in particular, could represent a better option to rapidly obtain potentially useful quantitative information and distinguish samples in larger datasets in combination with chemometric analysis.

### 3.2. Biological Activity

In the allelopathy and brine shrimp lethality tests, rosemary hydrosol appeared more toxic than sage and cypress extracts. This effect could be related, albeit partially, to the presence of 1,8-cineole, which was already described as a root growth inhibitor [35]; 1,8-cineole represented the main constituent not only of rosemary but also of sage hydrosol. Other constituents present in rosemary hydrosol should therefore contribute, possibly acting synergistically with 1,8-cineole, to its stronger allelopathy activity and brine shrimp toxicity compared to the sage hydrosol. It is worthy to note that the overall amount of monoterpene hydrocarbons is much higher in rosemary than sage hydrosol (15.6% and 1.3%, respectively); moreover, only 86.7% of the compounds in rosemary hydrosol were identified, while in sage, nearly all of them were determined (99.6%). The presence of higher amounts of monoterpenes or even other unidentified compounds could be responsible for the higher toxicity of rosemary hydrosol compared to sage. Concerning the antifungal activity, the presence of 1,8-cineole, camphor, and camphene in rosemary and sage hydrosols could be related, albeit partially, to their efficacy compared to the cypress hydrosol as mycostatic agents [36,37].

Considering the analysis on the hypothalamic cell line (Hypo-E22), only the sage hydrosol showed a viability reduction that appears concentration-dependent; this result could be due to the presence of α- and β-thujone, which are known to be neurotoxic derivatives [38]. Moreover, rosemary and sage were more effective than cypress in blunting the hydrogen peroxide-induced toxicity, thus restoring the basal viability levels. Particularly, the observed protective effects on hypothalamic cells occurred at hydrosol concentrations below the cytotoxic concentration (100 µg/mL) observed in the HCT116 cell line. These results, combined together, suggest that the rosemary hydrosol appears to be the best recommended extract at the neuronal level, being at the same time non-toxic in the concentration range of 5–50 µg/mL for hypothalamic cells and protective against hydrogen peroxide-induced toxicity. This is in line with the data on the beneficial properties on cognition and the cerebrovascular system of rosemary aromatic water observed in healthy adults [14].

Regarding the bioinformatics analysis, TRPM8, TRPV1, and VR1 were highlighted by the platform as putative targets with high scores (0.916–0.918). These receptors were detected in both the hypothalamus and colon. In the colon, they could mediate anti-proliferative and anti-inflammatory effects [39,40,41], thus supporting the observed cytotoxic effects on HCT116 cells, especially at the highest tested concentrations. In the hypothalamus, these receptors are mainly involved in neuromodulatory and neuroendocrine effects [42,43,44,45]. In this regard, we hypothesize that the hydrolates’ neuroprotective effects towards Hypo-E22 could be due, albeit partially, to their intrinsic antiradical effects.

## 4. Materials and methods

### 4.1. Chemicals

UHPLC-grade acetonitrile, formic acid, and water were purchased from Romil-Deltek (Italy). Borneol, camphor, 1,8-cineole, linalool, α-terpineol, 4-terpineol, α- and β-thujone, and verbenone, used as reference standards for UHPLC-MS analyses, were purchased from Sigma-Aldrich (Milano, Italy).

### 4.2. Plant Cultivation and Samples Preparation

Cypress (*Cupressus sempervirens* L.), rosemary (*Salvia rosmarinus* Spenn. syn. *Rosmarinus officinalis* L.), and sage (*Salvia officinalis* L.) were grown following biodynamic agricultural principles [46] in the agro-farm “Le Tassinaie”, which consists of a main house surrounded by 3 hectares of land mostly dedicated to agricultural activities. This agro-farm is located in the territory of Castellina Marittima within the district of Pisa in the Tuscany region, Italy. The woody cones of cypress (ca. 7 kg) and the flowering aerial parts of rosemary and sage (both ca. 3 kg) were manually collected in February, April, and June 2020, respectively. The fresh plant materials were distilled using the TredTechnology extractor of essential oils, model EOE20. In all cases, the plant materials were immersed in 4 L of tap water and distilled under vacuum at 0.4 bar and 85 °C for 1 h, obtaining in all cases around 1 L of the corresponding aromatic water; the corresponding essential oils obtained during the distillation process were removed from the top of the aromatic water with a glass syringe. It is important to note that these distillation conditions were optimized in order to obtain the aromatic waters as main products of such a manufacturing procedure, rather than as by-products, as discussed in-depth in a previous work [8].

### 4.3. HS-SPME-GC/MS Analysis

An SPME device coated with polydimethylsiloxane (PDMS, 100 μm) was obtained from Supelco. The fiber, conditioned according to the manufacturer’s recommendations, was exposed to the headspace of the hydrosols for 30 s, withdrawn into the needle and immediately transferred to the injection port of the GC/MS, where the fiber was desorbed with a splitless injection method. The gas chromatograph was an Agilent 7890B equipped with an Agilent HB-5MS (Agilent Technologies Inc., Santa Clara, CA, USA) capillary column (30 m × 0.25 mm; coating thickness 0.25 µm). The analytical conditions were as follows: injector and transfer line temperatures were 250 °C and 240 °C, respectively; the oven temperature was programmed from 60 to 240 °C at 3 °C/min; carrier gas helium was set at 1 mL/min. The gas chromatograph–electron impact mass spectrometer (GC–EIMS) was an Agilent 5977B single quadrupole mass detector (Agilent Technologies Inc., Santa Clara, CA, USA). Acquisition was performed in full scan within a 30–300 *m*/*z* range, with a scan time of 1.0 s.

### 4.4. NMR Analysis

^1^H-NMR spectra were obtained on a Bruker Avance III 400 MHz spectrometer. Rosemary, sage, and cypress samples were prepared by adding 60 μL of D_2_O as an internal lock to 600 μL of each hydrolate. Pre-saturation was carried out with a relaxation delay (d1 = 2 s) and mixing time (d18 = 0.8 s). In both cases, the number of scans was 64, and partial suppression of the solvent signals around 4.80 ppm was achieved.

### 4.5. UHPLC-HR-ESI-MS/MS Analysis

The UHPLC-HR-MS system was composed of a Vanquish Flex Binary pump LC and a Q Exactive Plus MS equipped with an electrospray ionization (ESI) source, the Orbitrap-based FT-MS system (Thermo Fischer Scientific Inc., Dreieich, Germany). The chromatography was performed on a Kinetex Biphenyl column (100 × 2.1 mm, 2.6 µm particle size) composed of SecurityGuardTM Ultra Cartridges (Phenomenex, Bologna, Italy). Elution was carried out with formic acid in H_2_O 0.1% *v*/*v* (solvent A) and formic acid in acetonitrile 0.1% *v*/*v* (solvent B) with a linear solvent gradient (5 to 60% B within 16 min). Samples (5 µL) were injected into the LC system at a flow rate of 0.5 mL/min, maintaining autosampler and column oven temperatures at 4 and 35 °C, respectively. MS parameters were applied as previously reported [8].

### 4.6. Biological Analysis

#### 4.6.1. Allelopathy Test

Commercial seeds of three lettuce varieties, namely Romana, Lollo Bionda, and Canasta, were selected for the test due to their fast germination rate and sensitivity. According to the method detailed in our previous paper [47], seeds were distributed on paper beds in a Petri capsule imbibed with diluted hydrosols (dilution [*v*/*v*] in distilled water 1/10 and 1/100). The emergence of at least 2 mm length with the typical geotropic curvature of the radicle was used as the criterium to define the presence/absence of germination. The seeds that showed false germination by soaking were not accounted for. The germination was recorded at 48, 72, and 96 h by counting the number of germinated seeds and were expressed as the mean value of at least triplicate experiments.

#### 4.6.2. Brine Shrimp Lethality Test

The cytotoxicity limits of the extracts in the range of 0.1–20 mg/mL were evaluated through the brine shrimp *Artemia salina* lethality bioassay, as previously reported [47]. Briefly, cysts of *Artemia salina* were hatched in artificial seawater (1 g cysts/L). After 24 h, living brine shrimp were distributed in a 6 well-plate in the presence of diluted plant hydrosols (500–10 µL/mL) in artificial seawater, and 24 h later, the living/dead organisms were monitored. The experiments were carried out in triplicate.

#### 4.6.3. Antifungal Activity

The in vitro antifungal activity of hydrosols were assessed against different yeasts, dermatophyte, and fungal pool species: *Candida albicans* (YEPGA 6138), *C. tropicalis* (YEPGA 6184), *Arthroderma crocatum* (CCF 5300), *A. gypseum* (CCF 6261), *A. quadrifidum* (CCF 5792), *Trichophyton mentagrophytes* (CCF 4823), *T. rubrum* (CCF 4879), *T. erinacei* (CCF 5830), and *T. tonsurans* (CCF 4834), as previously described [48].

#### 4.6.4. Cell Cultures, Viability Test, and Neuro-Protective Effects

The effects of the hydrosols (50–500 µL/mL) were preliminarily tested on the human HCT116 cell line (colorectal epithelial carcinoma), and the viability was measured through the 3-(4,5-dimethylthiazol-2-yl)-2,5-diphenyltetrazolium bromide (MTT) test in order to define the cell susceptibility. The detailed experimental conditions were reported in our previous paper [49]. In order to better elucidate a possible effect on the nervous system, a deeper investigation of the potential effects of subtoxic dilutions of hydrosols was conducted on non-tumor hypothalamic rat cells (Hypo-E22). Samples were tested at concentrations ranging from 5 to 50 µL/mL, and the biocompatibility was assessed through the MTT cell viability test. The highest concentration that was biocompatible was used to investigate protective effects against the neurotoxicity induced by hydrogen peroxide (300 µM). The detailed experimental conditions are reported in our previous paper [50]. Viability data were expressed as variation compared to the control untreated group.

#### 4.6.5. Inhibition of Horseradish Peroxidase

The activity of horseradish peroxidase (HRP) can be monitored through the de-coloration of the phenol red used as substrate. Each hydrosol was directly tested on a 96-well flat-bottom microplate containing test sample (10 μL), 10 nM peroxidase enzyme solution (5 μL), 50 μM phenol red (5 μL), and 50 mM pH 7.4 phosphate buffer solution (170 μL). After incubation for 5 min in the dark at room temperature, 50 μM hydrogen peroxide (10 μL) was added. After 10 min, the absorbance was recorded at λ = 550 nm, and the percentage inhibition was calculated.

#### 4.6.6. In Silico Analysis

Putative targets were identified according to the bioinformatics method recently described by Gu and colleagues [51]. Briefly, proteins targeted by extracts were predicted using the bioinformatics platform STITCH; the same resource was employed for the network-pharmacology and gene ontology analyses.

#### 4.6.7. Statistical Analysis

Statistical analysis was performed using GraphPad Prism version 5.01 for Windows (GraphPad Software, San Diego, CA, USA). Means ± S.E.M. were determined for each experimental group and analyzed by one-way analysis of variance (ANOVA), followed by Tukey test. Statistical significance was set at *p* < 0.05.

## 5. Conclusions

In the present study, a multidirectional approach was conducted to unravel the phytochemical composition and biological properties of the hydrosols from sage, rosemary, and cypress. Monoterpenes and especially oxygenated monoterpenes represent the most abundant classes in all samples. In the case of rosemary, the presence of 1,8-cineole, camphor, and camphene could be at the basis of its high activity as an antimycotic agent compared to sage and cypress hydrosols. On the other hand, the levels of 1,8-cineole and camphor in rosemary and sage could also be responsible for the higher potency as cytotoxic agents compared to cypress, whereas the very close protective effects exerted by all three hydrosols could be partly due to their intrinsic antiradical properties. The overall experience with these extracts indicates the hydrolates are an important source of relevant phytochemicals with significant pharmacological potential.

On the basis of experimental data, we can speculate a wider range of potential applications for hydrosols, ranging from weed management to the formulation of hygiene-related products, such as for pool management or for body care. Regardless, some limitations of the present study, including the variability of the phytocomplex and hydrodistillation process (solvent/matrix ratio, temperature, pressure, time), suggest that future deeper investigations would be beneficial to confirm the rational base for these innovative applications.

## Figures and Tables

**Table 1:** Total volatile organic compound (VOC) profiles for hydrolates of rosemary (R), sage (S), and cypress (C) by HS-SPME-GC-MS.

			Relative Content %		
Compound	*t*_R_ (min)	LRI	R	S	C
Monoterpene hydrocarbons			15.6	1.3	23.0
α-pinene	5.61	939	1.4	0.1	4.5
camphene	5.94	953	3.9	0.1	-
thuja-2,4(10)-diene	6.05	957	0.9	-	-
sabinene	6.55	976	-	-	2.4
β-pinene	6.69	980	3.6	0.8	0.2
myrcene	7.03	991	1.9	-	0.9
α-phellandrene	7.47	1005	0.4	-	0.2
δ-3-carene	7.66	1011	0.4	-	4.9
α-terpinene	7.87	1018	0.6	-	0.9
*p*-cymene	8.09	1026	-	-	1.4
limonene	8.23	1031	-	-	2.4
γ-terpinene	9.30	1062	1.4	0.2	1.4
terpinolene	10.34	1088	1.1	0.1	3.8
Oxygenated monoterpenes			70.4	93.7	73.9
1,8-cineole (eucalyptol)	8.64	1033	47.1	42.9	1.1
linalool	10.83	1098	2.0	-	0.4
filifolone	10.95	1108	1.6	-	-
α-thujone	11.13	1110	-	24.3	0.4
exo-fenchol	11.26	1112	-	-	0.2
β-thujone	11.53	1114	-	14.7	0.5
*cis*-*p*-menth-2-en-1-ol	11.57	1121	-	-	1.6
α-campholenal	11.77	1125	0.2	-	-
*trans*-pinocarveol	12.24	1139	0.2	-	-
*trans*-*p*-menth-2-en-1-ol	12.27	1140	-	-	0.9
camphor	12.58	1143	5.4	8.9	1.1
camphene hydrate	12.63	1148	-	-	0.3
*trans*-pinocamphone	13.15	1160	0.2	-	-
pinocarvone	13.24	1162	0.6	-	-
borneol	13.44	1165	3.7	1.9	1.6
*cis*-pinocamphone	13.74	1173	1.0	-	-
4-terpineol	13.85	1177	0.7	0.6	44.5
α-terpineol	14.38	1190	0.8	0.2	3.4
*cis*-piperitol	14.55	1193	-	-	0.3
myrtenol	14.62	1193	0.2	-	-
*trans*-piperitol	15.05	1205	-	-	0.1
verbenone	15.20	1206	2.8	-	0.1
methyl carvacrol	16.59	1244	-	-	0.3
piperitone	16.98	1252	-	-	0.4
geraniol	17.04	1255	0.4	-	-
bornyl acetate	18.40	1285	3.4	0.2	3.5
γ-terpinyl acetate	20.60	1341	-	-	2.6
α-terpinyl acetate	20.99	1350	-	-	10.6
geranyl acetate	22.44	1383	0.1	-	-
Sesquiterpene hydrocarbons			0.7	4.3	2.3
α-cubebene	20.98	1351	-	0.1	-
α-copaene	22.06	1376	-	0.2	-
α-cedrene	23.55	1409	-	-	0.7
β-caryophyllene	23.84	1418	0.6	2.7	0.7
aromadendrene	24.61	1439	-	0.2	-
α-humulene	25.21	1454	0.1	0.6	0.2
γ-murolene	26.15	1477	-	0.2	-
germacrene D	26.30	1480	-	-	0.6
valencene	26.86	1491	-	0.1	-
δ-cadinene	27.99	1524	-	0.2	-
Oxygenated sesquiterpenes			-	0.3	0.6
caryophyllene oxide	30.23	1581	-	0.1	-
viridiflorol	30.56	1590	-	0.2	-
cedrol	30.91	1596	-	-	0.6
Total identified			86.7	99.6	99.7

**Table 2:** Chromatographic (*t*_R_, retention time) and HR-ESI–MS/MS data of compounds identified in hydrolates of rosemary (R), sage (S), and cypress (C).

Compound ^a^	*t*_R_ (min)	Precursor Ion (*m*/*z*)	HR(+ESI)-MS/MS ^b^ Product Ions (*m*/*z*)	Molecular Formula	Exper. Mass	Theor. Mass	Error (ppm)	Hydrolate
Verbenone	12.4	151.1116 ([M+H]^+^)	123.08, **109.07**	C_10_H_14_O	151.1116	151.1117	−0.66	R, C
Borneol	13.6	137.1324 ([M+H-H_2_O]^+^)	109.10, 95.09, **81.07**	C_10_H_18_O	137.1324	137.1325	−0.73	C
Linalool	13.8	137.1323 ([M+H-H_2_O]^+^)	109.10, **95.09**, 81.07	C_10_H_18_O	137.1323	137.1325	−1.46	R, C
4-Terpineol/α-Terpineol	13.9	137.1323 ([M+H-H_2_O]^+^)	109.10, **95.09**, 81.07	C_10_H_18_O	137.1323	137.1325	−1.46	S, C
Camphor	14.1	153.1271 ([M+H]^+^)	135.12, 109.10, **97.07**	C_10_H_16_O	153.1271	153.1274	−1.96	R, S
α/β-Thujone	14.2	153.1272 ([M+H]^+^)	135.12, 109.10, **97.07**	C_10_H_16_O	153.1272	153.1274	−1.31	S
1,8-Cineole (Eucalyptol)	14.7	137.1323 ([M+H-H_2_O]^+^)	109.10, 95.09, **81.07**	C_10_H_18_O	137.1323	137.1325	−1.46	R, S
Piperitone	14.8	153.1271 ([M+H]^+^)	135.12, 109.10, 97.07, **81.07**, 71.05	C_10_H_16_O	153.1272	153.1274	−1.31	C

^a^ Compounds are listed by elution order; all compounds were identified by comparison of data (full MS, MS pathway fragmentation, and *t*_R_) with those of reference standards, except for piperitone, which was tentatively identified. ^b^ The base ion peak is in bold.

**Table 3:** Minimal inhibitory concentrations (MICs) of aromatic waters against yeast and dermatophyte strains.

	MIC *								
	*Trichophyton*	*Trichophyton*	*Trichophyton*	*Trichophyton*	*Arthroderma*	*Arthroderma*	*Arthroderma*	*Candida*	*Candida*
	*mentagrophytes*	*tonsurans*	*rubrum*	*erinacei*	*crocatum*	*quadrifidum*	*gypseum*	*albicans*	*tropicalis*
Fungal Strain	(CCF 4823)	(CCF 4834)	(CCF 4879)	(CCF 5930)	(CCF 5300)	(CCF 5792)	(CCF 6261)	(YEPGA 6138)	(YEPGA 6184)
Hydrosol									
Cypress	99.2	39.4	397.0	397.0	24.8	39.4	198.4	49.6	78.7
	(62.5–125)	(31.3–62.5)	(250–500)	(250–500)	(15.6–31.3)	(31.3–62.5)	(125–250)	(31.3–62.5)	(62.5–125)
Rosemary	12.4	19.7	24.8	315.0	39.4	24.8	157.5	19.7	24.8
	(7.8–15.6)	(15.6–31.3)	(15.6–31.3)	(250–500)	(31.3–62.5)	(15.6–31.3)	(125–250)	(15.6–31.3)	(31.3–62.5)
Sage	19.7	24.8	24.8	397.0	49.6	99.2	315.0	19.7	24.8
	(15.6–31.3)	(15.6–31.3)	(15.6–31.3)	(250–500)	(31.3–62.5)	(62.5–125)	(250–500)	(15.6–31.3)	(15.6–31.3)
Fluconazole	>16	2	8	>16	8	>16	>16	2	4
Griseofulvin	1	0.125	2	0.25	>8	>8	>8	>8	>8

* MIC values are reported as geometric means of 3 independent replicates (*n* = 3). MIC range concentrations (µL/mL) are reported within brackets. MIC values of reference drugs are expressed as µg/mL.

**Table 4:** Inhibition of horseradish peroxidase (HRP).

	Hydrosol		
	Cypress	Rosemary	Sage
HRP % inhibition	25.94 ± 1.84	19.53 ± 1.58	31.58 ± 2.04

## Supplementary Materials

The following supporting information can be downloaded at: https://www.mdpi.com/article/10.3390/plants11030349/s1, Figure S1: GC-MS chromatogram of rosemary hydrosol headspace; Figure S2: GC-MS chromatogram of sage hydrosol headspace; Figure S3: GC-MS chromatogram of cypress hydrosol headspace.
