# Supplementary material for: Hydrosols from Rosmarinus officinalis, Salvia officinalis, and Cupressus sempervirens: Phytochemical Analysis and Bioactivity Evaluation
Source: Plants (Basel). 2022 Jan 27;11(3):349. doi: 10.3390/plants11030349 (PMC8840401; doi:10.3390/plants11030349)
Supplement: Supplementary file 1 [file plants-11-00349-s001.zip › plants-1524785-supplementary.pdf]

Supplementary Material

Hydrosols from *Rosmarinus officinalis, Salvia officinalis,* and *Cupressus sempervirens:* Phytochemical analysis and bioactivity evaluation

Matteo Politi [1], Claudio Ferrante [1], Luigi Menghini [1], Paola Angelini [2], Giancarlo Angeles Flores [2], Beatrice Muscatello [3,4], Alessandra Braca [3,4,*] and Marinella De Leo [3,4]

[1] Dipartimento di Farmacia, Botanic garden "Giardino dei Semplici", Università di Chieti-Pescara, via Vestini 1, 66100, Chieti Scalo, Italy; matteo.politi@unich.it (M.P.); claudio.ferrante@unich.it (C.F.); luigi.menghini@unich.it (L.M.)

[2] Dipartimento di Chimica, Biologia e Biotecnologia, Università di Perugia, Via del Giochetto 6, 06122 Perugia, Italy; paola.angelini@unipg.it (P.A.); giancarlo.angelesflores@studenti.unipg.it (G.A.F.);

[3] Dipartimento di Farmacia, Università di Pisa, via Bonanno 33, 56126, Pisa, Italy; beatrice.muscatello@unipi.it (B.M.); alessandra.braca@unipi.it (A.B.); marinella.deleo@unipi.it (M.D.L.)

[4] CISUP, Centro per l'Integrazione della Strumentazione dell'Università di Pisa, Lungarno Pacinotti 43, 56126 Pisa, Italy

* Correspondence: alessandra.braca@unipi.it (A.B.); Tel: +39-050-2219688

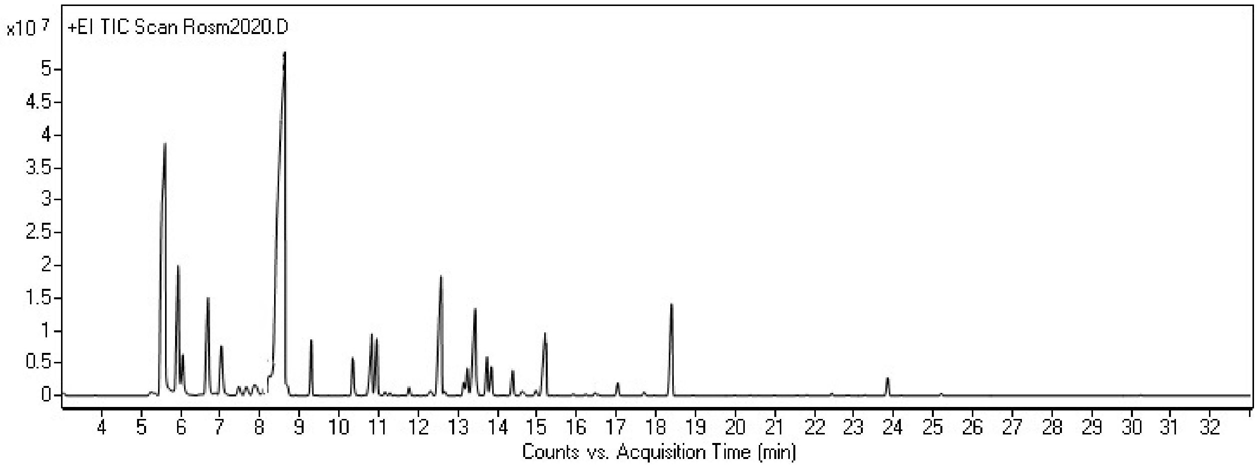

Figure S1. GC-MS chromatogram of rosemary hydrosol headspace.

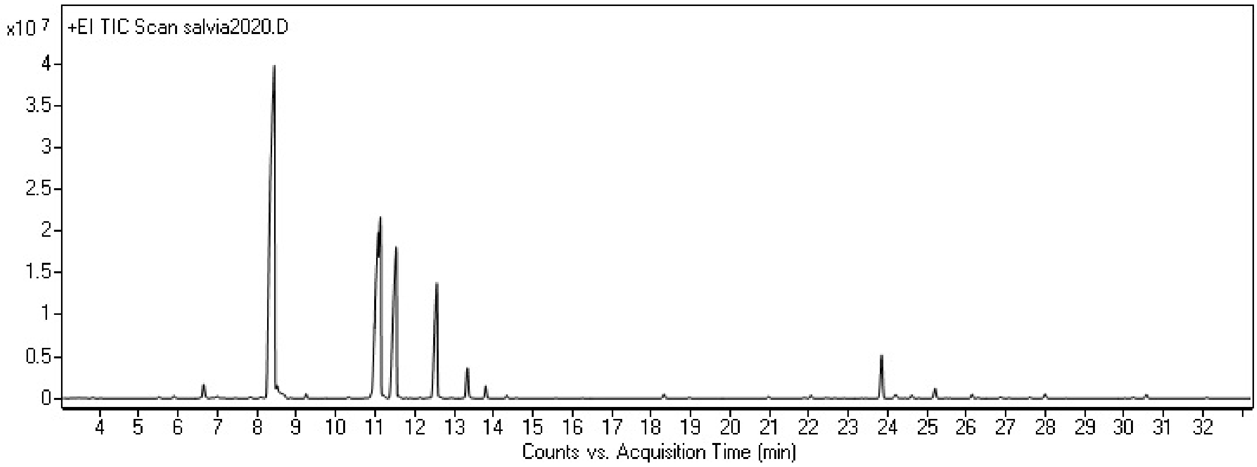

Figure S2. GC-MS chromatogram of sage hydrosol headspace.

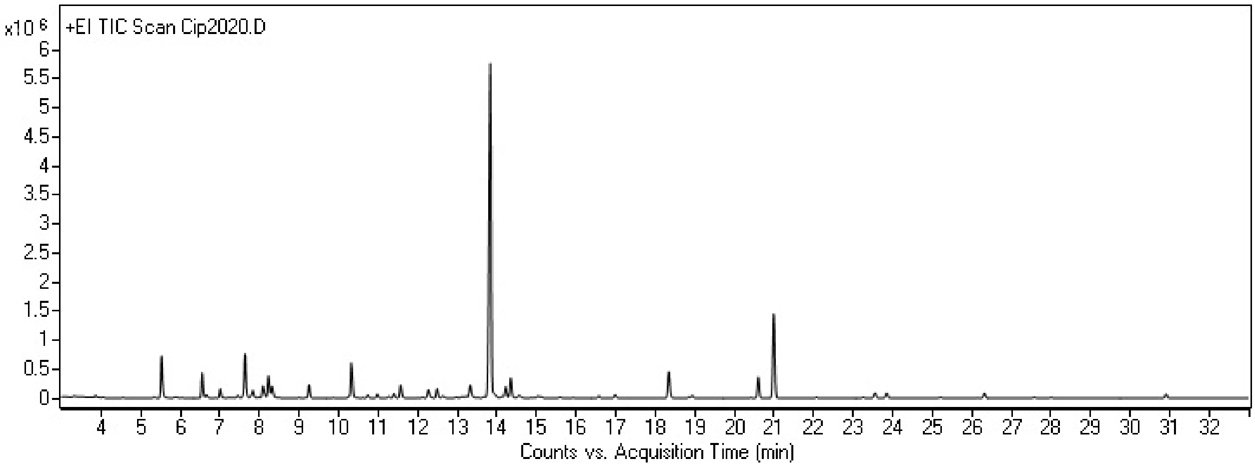

Figure S3. GC-MS Chromatogram of cypress hydrosol headspace.
